# Society Meetings

**Published:** 1893-12

**Authors:** 


### Society Meetings.

The Tri-State Medical Society (Alabama, Tennessee, and Georgia) held one of the most interesting meetings in its history, at Chattanooga, in October. A very full account of the proceedings has been published, and advance sheets have been sent out to the journals. We regret that we have not space to give an abstract of the work done at the meeting. It is proposed to hold the next meeting in Atlanta on the second Tuesday of October, 1894, and there is a proposition on the table to change its name to the Southeastern Medical Society, which will be considered at that time. This will embrace the territory east of the Mississippi and south of the Ohio. We think it is well, in view of the fact that there are several of these triple State organizations, for them to adopt more distinctive names.

---

The American Electro-Therapeutic Association, at its last meeting, elected, for the ensuing year, the following-named officers: Dr. W. J. Herdman, of Ann Arbor, president; Dr. Margaret Cleaves, of New York, secretary; Dr. Franklin H. Martin, of Chicago, and Dr. A. Lapthorn Smith, of Montreal, vice-presidents; Dr. R. J. Nunn, of Savannah, Ga., treasurer. It was decided to hold the next meeting in New York City on the last Tuesday in September, 1894.

---

The Eleventh International Medical Congress.—Dr. A. Jacobi, chairman of the American National Committee of the International Medical Congress, has issued a circular, in which he says that this Congress, which was postponed from September 24th, on account of cholera prevailing in Italy, according to a notification that he has received from the Secretary-General, will be held at Rome from March 29 to April 5, 1894. Instructions and documents relating to the journey, etc., are promised for the near future.

